# The Beneficial Effect of Swimming Training Associated with Quercetin Administration on the Endothelial Nitric Oxide-Dependent Relaxation in the Aorta of Rats with Experimentally Induced Type 1 Diabetes Mellitus

**DOI:** 10.3390/metabo13050586

**Published:** 2023-04-24

**Authors:** Irina-Camelia Chis, Carmen-Maria Micu, Alina Toader, Remus Moldovan, Laura Lele, Simona Clichici, Daniela-Rodica Mitrea

**Affiliations:** 1 Department of Physiology, Iuliu Hatieganu University of Medicine and Pharmacy, 1-3 Clinicilor Street, 400006 Cluj-Napoca, Cluj County, Romania; ichis@umfcluj.ro (I.-C.C.); toader.alina@umfcluj.ro (A.T.); moldovan.remus@umfcluj.ro (R.M.); rdmitrea@gmail.com (D.-R.M.); 2 Department of Anatomy and Embryology, Iuliu Hatieganu University of Medicine and Pharmacy, 3-5 Clinicilor Street, 400006 Cluj-Napoca, Cluj County, Romania; carmen.micu@umfcluj.ro; 3 Department of Medical Disciplines, Faculty of Medicine and Pharmacy, University of Oradea, 10 1 Decembrie Street, 410073 Oradea, Bihor County, Romania; dr.laura.lele@gmail.com

**Keywords:** acetylcholine, endothelium, diabetes, quercetin, moderate swimming training, aorta

## Abstract

Type 1 diabetes mellitus is related to the vascular oxidative and nitrosative stress, the trigger for atherosclerosis and cardiovascular complications. The effects of moderate swimming training associated with quercetin oral administration were evaluated in aorta of rats with experimentally induced type 1 diabetes mellitus (T1DM), by analysing the nitric oxide-endothelial dependent relaxation (NO-EDR). T1DM rats received daily quercetin 30 mg/kg and followed the protocol of 5-weeks swimming exercise (30 min/day; 5 days/week). Aorta relaxation to acetylcholine (Ach) and sodium nitroprusside (SNP) were measured at the end of the experiment. Ach-induced endothelial dependent relaxation was significantly decreased in phenylephrine (PE) pre-contracted aorta of diabetic rats. Swimming exercise with quercetin administration preserved Ach-induced EDR but did not have any impact on SNP-induced endothelium-independent relaxation in the diabetic aorta. These findings suggest that quercetin administration associated with moderate swimming exercise could improve the endothelial NO-dependent relaxation in the aorta of rats with experimentally induced type 1 diabetes mellitus, showing that this therapeutical combination may improve and even prevent the vascular complications that occur in diabetic patients.

## 1. Introduction

The endothelial dysfunction (ED) that occurs in type 1 diabetes mellitus (T1DM) involves numerous and diverse pathogenetic mechanisms including inflammation, hyperglycaemia, production of reactive oxygen species (ROS) and of reactive nitrogen species (RNS), the decrease of nitric oxide (NO) availability or dyslipidemia [1,2,3,4,5,6,7,8,9]. Endothelial dysfunction is characterised by altered endothelial-dependent relaxation (EDR) produced by the decreased NO levels in the vessel wall [8,9,10], a process that leads to micro- and macrovascular complications, increasing significantly the morbidity and mortality in T1DM [1].

Endothelial dysfunction decreases the EDR to vasorelaxant substances like acetylcholine (Ach) and represents a preliminary phase in the development of vascular alterations. ED development is based on oxidative stress and ROS production, especially of the superoxide anion, which has an important role in NO scavenging. 

The moderate training physical effort enhances the insulin sensitivity and the carbohydrates metabolism in T1DM. Several recent studies made on T1DM presented the ameliorated endothelial dysfunction and the improved EDR after moderate exercise training that decreased the inflammation, the ROS/RNS production, and increased NO levels in vessel walls [4,6,11,12,13,14]. The type of the performed physical effort and its duration were correlated with the training efficacy in T1DM [11], the favourable effects in EDR enhancement and on the oxidative/nitrosative stress being recorded only after moderate exercise training [15] while the strenuous training intensified the ROS production [11,15].

Numerous studies present the flavonoids like potent natural antioxidants in diabetes mellitus, preventing the cardiovascular complications [16,17]. 

Quercetin is a flavonol of plant origin found in numerous vegetables and fruits like onion, tea, grapes, apple, berries, etc. [18]. Quercetin presents beneficial effects: antioxidant, hypoglycaemic, vasodilator, anti-inflammatory, antiapoptotic, anti-atherogenic, hypolipidemic, etc. [18,19,20,21,22,23,24]. It is a flavonol that protects the function and the viability of β-pancreatic cells against oxidative stress and may stimulate the insulin secretion regenerating the islets of Langerhans [25]. Quercetin is an efficient antioxidant, being able to scavenge directly the ROS and to increase the endogenous antioxidants [18,21]; it may improve the endothelial function in hyperglycaemic conditions through its vasodilator endothelium-dependent effects, protecting the eNOS levels, decreasing the iNOS and enhancing the NO bioavailability [4,21,26].

In the present study, the effects on endothelium dysfunction of moderate exercise training associated with quercetin administration in an experimentally induced type 1 diabetes mellitus were investigated. Since the Ach-induced EDR was affected in aorta at 6 weeks after diabetes mellitus development, the authors hypothesised that moderate swimming training associated with oral administration of quercetin may restore the EDR.

## 2. Material and Methods

### 2.1. Drugs and Chemicals

Streptozotocin (STZ), phenylephrine (PE), indomethacin (IND), acetylcholine chloride (Ach), sodium nitroprusside (SNP), and quercetin (Que) were all obtained from Sigma-Aldrich Chemical Company Inc. (Gillingham, Dorset, UK). Streptozotocin was freshly dissolved in citrate sodium buffer (0.1 mol/L, pH 4.5) and maintained on ice before being used. Quercetin was suspended in 0.5% carboxymethyl cellulose (CMC) solution as a vehicle.

### 2.2. Equipment

Biopac MP150, modular tissue baths DA 100C, UgoBasile, Trappe, PA, USA was used to analyse the aorta rings responses at different solutions.

### 2.3. Procedure

The study was realised in the Physiology Department of Iuliu Hatieganu University of Medicine and Pharmacy Cluj-Napoca Romania, with the approval of Ethical Committee on Animal Welfare (No. 44/13.03.2017) of A.N.S.V.S.A. (The National Sanitary Veterinary and Food Safety Authority) and following the Guidelines in the Use of Animals in Toxicology. 

#### 2.3.1. Experimental Protocol

Male albino Wistar rats, with ages of three months, weights between 260 and 310 g, were purchased from the Biobase of Iuliu Hatieganu University of Medicine and Pharmacy, Cluj-Napoca, Romania. The animals were fed with a standard diet, water ad libitum, and were kept in standard conditions (22 ± 2 °C, 45–50% relative humidity) with a day–night cycle of 12 h. 

For induction of type 1 diabetes mellitus, streptozotocin (STZ) 60 mg/kg intraperitoneal single injection was used. Carboxymethyl cellulose was used for streptozotocin solution [4,20,27]. After 96 h post-injection, all the rats developed T1DM which was confirmed through the fasting blood glucose levels that were above 250 mg/dL (13.89 mmol/L). After 7 days from the T1DM development, exercise training was started in association with quercetin (Que) administration (30 mg/kg body weight/day/5 weeks. The animals from the control group received carboxymethyl cellulose (CMC) 0.6 mL/day for 5 weeks. 

**Figure 1:**
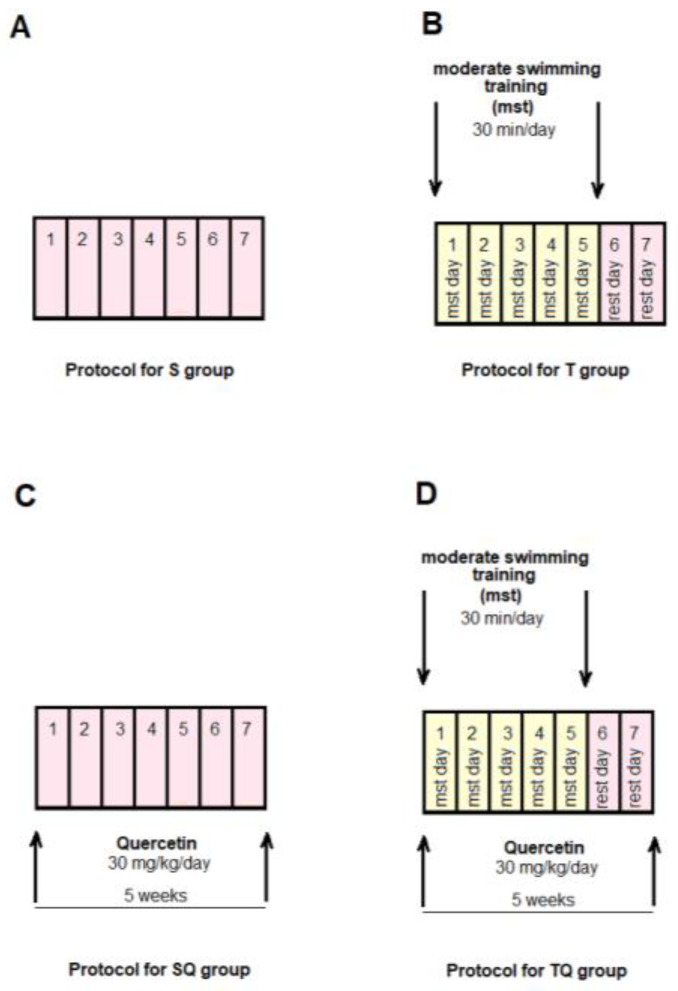
The time course of scheduled experiment for the groups of rats without T1DM: (**A**) healthy sedentary rats (S group); (**B**) healthy trained rats, 30 min/day, 5 days/week for 5 weeks (T group); (**C**) healthy sedentary rats treated with quercetin 30 mg/kg/day, for 5 weeks (SQ group); (**D**) healthy trained rats, 30 min/day, 5 days/week and treated with quercetin 30 mg/kg/day, for 5 weeks (TQ group).

**Figure 2:**
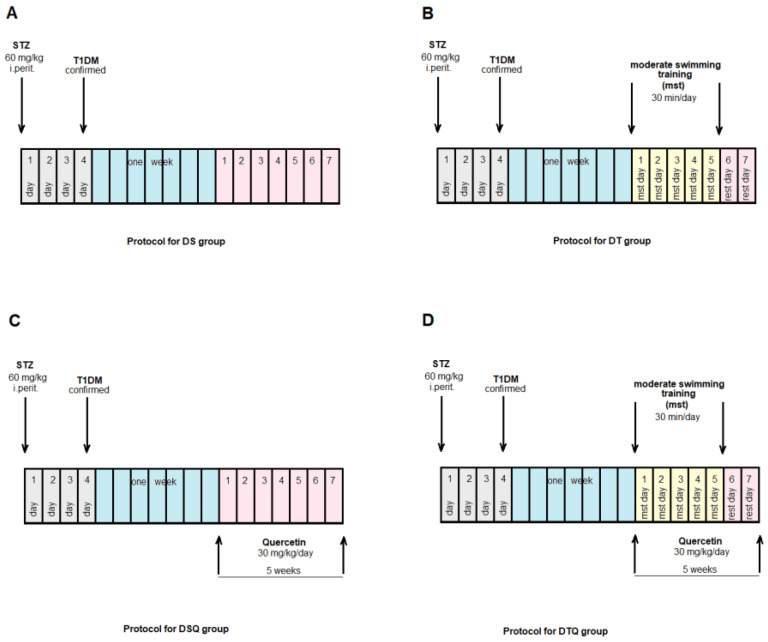
The time course of scheduled experiment for the groups of rats with T1DM: (**A**) diabetic sedentary rats (DS group); (**B**) diabetic trained rats, 30 min/day, 5 days/week for 5 weeks (DT group); (**C**) diabetic sedentary rats treated with quercetin 30 mg/kg/day, for 5 weeks (DSQ group); (**D**) diabetic trained rats, 30 min/day, 5 days/week and treated with quercetin 30 mg/kg/day, for 5 weeks (DTQ group).

The animals were randomly allocated into four control groups (N = 10): Group S-sedentary, untreated animals; Group T-trained, untreated animals; Group SQ-sedentary animals, treated with quercetin; Group TQ-trained animals, treated with quercetin (Figure 1); and into four experimental diabetic groups: Group DS-diabetic, sedentary, untreated animals; Group DT-diabetic, trained, untreated animals; Group DSQ-diabetic, sedentary animals, treated with quercetin; and Group DTQ-diabetic, trained animals, treated with quercetin (Figure 2).

At the end of the experiment, after the last night with food deprivation, the glycaemia was measured from retro-orbital venous plexus with the ACCU-CHEK Sensor System from Roche Diagnostics GmbH (Mannheim, Germany). Deep anaesthesia of all the rats was realised with ketamine 10% (5 mg/100 gbw) and xylazine hydroxychloride 2% (100 mg/100 gbw), to collect the samples of thoracic aorta while the connective tissue was removed.

#### 2.3.2. Moderate Swimming Training Protocol

The rats were trained through a moderate swimming exercise inside a cylindrical tank (60/100/45 cm) at a temperature maintained constantly at 36 °C. The swimming exercise was performed for 30 min/day, 5 days/week, for 5 weeks [4,28,29,30].

#### 2.3.3. Preparation of Aortic Tissue and Measurement of Isometric Force 

Thoracic aorta fragments between aortic arch and diaphragm were taken and inserted in ice-cold, oxygenated, modified Krebs–Henseleit solution (KHS) that consisted of (in mM) 118 NaCl; 25.0 NaHCO_3_; 4.7 KCl; 1.6 CaCl_2_; 1.2 KH_2_PO_4_; 1.2 MgSO_4_ and 11.1 Glucose. Thoracic aortas were segmented into fragments of 2–2.5 mm width. The aorta rings were placed between stainless steel triangles into individual tissue bath (Tissue Bath Station: for baths of 20 mL with MP150 Data Acquisition System-BIOPAC System Inc., Goleta, CA, USA), with oxygenated (95% O_2_, 5% CO_2_) modified KHS (37 °C, pH 7.4), one end connected to a tissue holder and the other to a force-displacement transducer (Isometric Force Transducers-BIOPAC System Inc., USA).

To evaluate the endothelial dysfunction, isolated vessel in tissue bath was performed using the experimental protocol for endothelial vasomotor response, and to analyse the vascular reactivity, cumulative doses of vasodilator agents were used. In the tissue bath, indomethacin (10^−5^ M) was added to inhibit the prostaglandin release. The aorta samples were maintained for 60 min at 1.5–2 g resting tension (the optimal tension obtained through prior experiments) with KHS solution changed every 20 min. 

The viability of the aorta smooth muscle was evaluated before the beginning of the vessel reactivity test by achieving two similar contractile responses at KCl, 80 mM/L, the contraction to KCl representing the standard of maximum contraction. The response through contraction to PE must be 80–100% from the KCl contraction. 

The vasodilator response to Ach (10^−5^ M) above 10–15% of the contraction produced by KCl ensures the viability of the endothelium. 

#### 2.3.4. Testing Phase: The Evaluation of Phenylephrine (PE), Acetylcholine (Ach), and Sodium Nitroprusside (SNP) Effects on Aorta Rings

The endothelial integrity was established, and then, the aorta rings were precontracted with phenylephrine (PE), an α_1_-adrenergic agonist, in increasing concentrations (10^−9^ to 10^−6^ M) in organ bath, until a stable plateau of the vessel smooth muscle tension was obtained. For all the investigated aorta rings, vasoconstrictor substance was added in cumulative concentrations inside the organ bath. The percentage of the KCl contraction was used to express the contractile response to PE. This test was done to investigate the maximum contraction of the aorta rings.

To evaluate the maximum endothelium-dependent relaxation of rats’ aortas, the aorta rings were pre-contracted with PE before being exposed to the cumulative concentrations of acetylcholine (10^−9^ to 10^−5^ M), an endothelium-dependent vasorelaxant. Subsequently, the aorta rings were washed with Krebs solution and then pre-contracted with a similar concentration of PE, before being treated with cumulative concentrations of SNP (10^−11^ to 10^−6^ M), an endothelium-independent vasorelaxant. This test was done to investigate the maximum endothelial-independent relaxation in the rats’ thoracic aortas.

#### 2.3.5. Statistical Analysis

The precontraction PE-induced was expressed as dose–response assessment calculated as percentage of contraction produced by high KCl solution/mg tissue weight against PE concentration (logarithmic scale). For the relaxation Ach-induced, the dose–response curves were obtained as a percentage of PE (0.1 μM) contraction/mg tissue weight against Ach concentration (logarithmic scale), while for the relaxation SNP-induced, the dose–response graphs were calculated as percentage of PE (0.1 μM) contraction/mg tissue weight against SNP concentration (logarithmic scale).

The relaxation, Ach- and SNP-induced, was calculated at every concentration as the percentage of relaxation from the maximum PE contraction. The contraction force was calculated as a percentage of maximum contraction at the highest dose of PE.

The statistical interpretation of the data was realised using Two-way ANOVA followed by the Post-test Bonferroni. The significance threshold was set at *p* < 0.05.

## 3. Results

### 3.1. Blood Glucose and Animal Body Weights in the Control and Experimental Groups

At the beginning of our study, all the rats had similar values of the fasting blood glucose (FBG) and of the body weights (BW). At 7 days after streptozotocin (STZ) administration, FBG levels increased significantly in diabetic groups (DS, DT, DSQ, DTQ), compared to the control groups (S, T, SQ, TQ). In comparison with DS group, moderate swimming training produced significant decreases of the FBG levels in DT group (*p* < 0.05), and when this type of exercise was associated with quercetin administration (DSQ group), a greater reduction (*p* < 0.0001) of this investigated parameter was recorded.

The body weights of rats did not present significant modifications among the groups, with one exception: sedentary diabetic rats (DS group) showed significant decreases (*p* < 0.05) compared to sedentary untreated control (S) group of rats (Table 1).

### 3.2. Aortic Contractile Responses to Phenylephrine (PE) in Control and Experimental Groups

**Figure 3:**
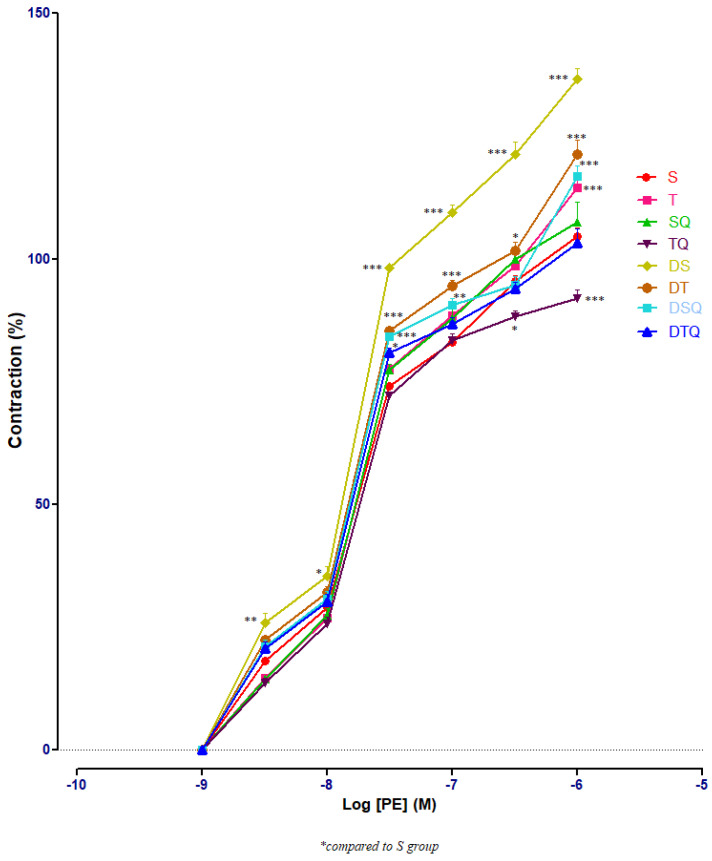
The aorta rings contractile response to cumulative concentrations of phenylephrine (PE) (10^−9^–10^−6^ M) in: sedentary, untreated animals (S); trained, untreated animals (T); sedentary animals, treated with quercetin (SQ); trained animals, treated with quercetin (TQ); diabetic, sedentary, untreated animals (DS); diabetic, trained, untreated animals (DT); diabetic, sedentary animals, treated with quercetin (DSQ) and in diabetic, trained animals, treated with quercetin (DTQ). Contraction was expressed as % of high KCl contraction. Values are expressed as mean ± SEM (* *p* < 0.05; ** *p* < 0.01; *** *p* < 0.001 compared to S group).

The responses of aorta rings to phenylephrine (PE) were investigated using cumulative doses (10^−^^9^ to 10^−^^6^ M). Sedentary diabetic rats (DS group) had significant increases of aorta rings contractile responses, compared to healthy sedentary (S group) or trained rats (T group) (*p* < 0.001), especially at PE between 3 × 10^−7^ and 10^−6^ M (not signalised in Figure 1 because of too many points of significance). The quercetin administration (DSQ group) or moderate swimming training associated with quercetin treatment (DTQ group) in diabetic rats decreased significantly (*p* < 0.001) the vessels’ contraction, compared to sedentary diabetic rats (group DS), between the same concentrations of PE (3 × 10^−7^–10^−6^ M) (Figure 3).

### 3.3. Aortic Relaxation Responses to Acetylcholine (Ach) in Control and Experimental Groups

**Figure 4:**
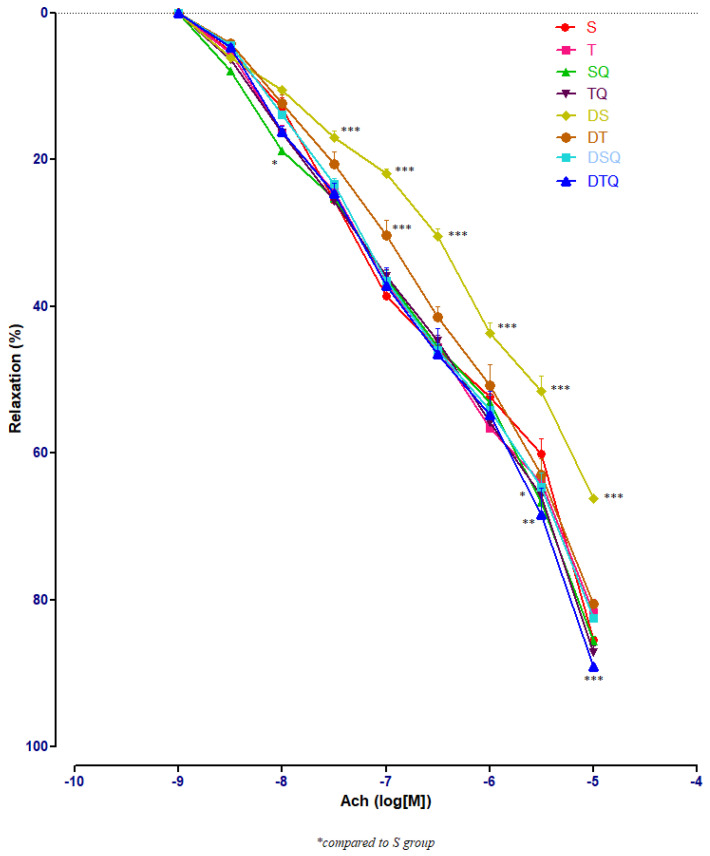
The aorta rings relaxation responses to cumulative concentrations of acetylcholine (Ach) (10^−9^–10^−5^ M) in: sedentary, untreated animals (S); trained, untreated animals (T); sedentary animals, treated with quercetin (SQ); trained animals, treated with quercetin (TQ); diabetic, sedentary, untreated animals (DS); diabetic, trained, untreated animals (DT); diabetic, sedentary animals, treated with quercetin (DSQ) and in diabetic, trained animals, treated with quercetin (DTQ). The relaxation responses are expressed as percentages of relaxation from an induced maximal contraction at PE. The values are expressed as mean ± SEM (* *p* < 0.05; ** *p* < 0.01; *** *p* < 0.001 compared to S group).

To determine the effects of the moderate swimming training associated or not with quercetin administration on endothelium-dependent relaxation, acetylcholine (Ach) of different concentrations (10^−9^–10^−5^ M) was used. The responses in the pre-contracted aorta rings of healthy and of diabetic rats were recorded. Figure 4 presents all the results of all the investigated groups; the numerous significant values are not marked on the graph but explained in the separated next images.

**Figure 5:**
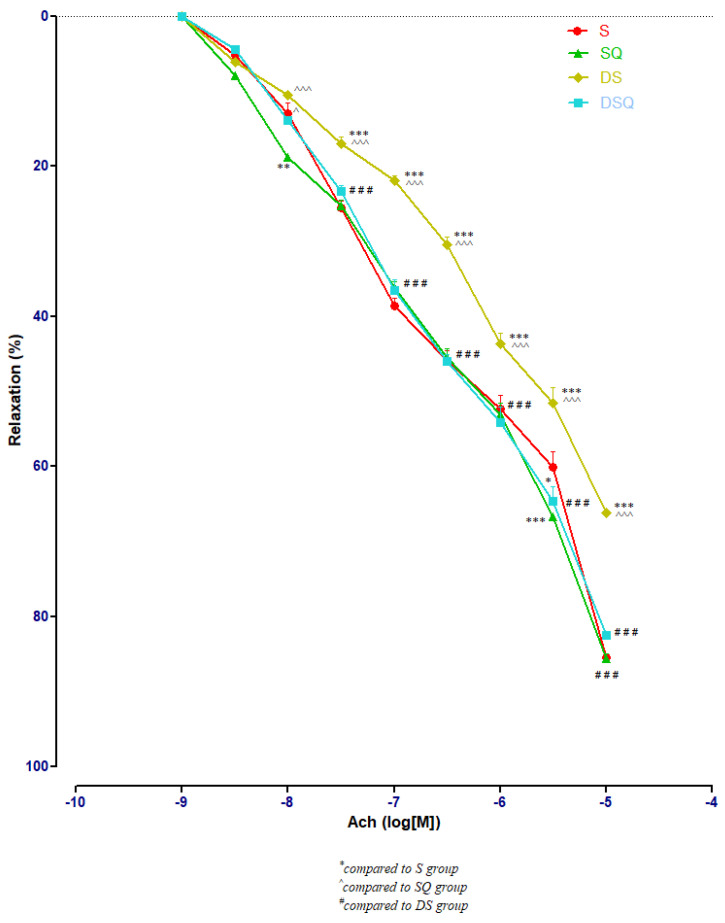
The aorta rings relaxation responses to cumulative concentrations of acetylcholine (Ach) (10^−9^–10^−5^ M) in: sedentary, untreated animals (S); sedentary animals, treated with quercetin (SQ); diabetic, sedentary, untreated animals (DS); diabetic, sedentary animals, treated with quercetin (DSQ). The relaxation responses are expressed as percentages of relaxation from an induced maximal contraction at PE. The values are expressed as mean ± SEM (* *p* < 0.05, ** *p* < 0.01, *** *p* < 0.001 compared to S group; ^ *p* < 0.05, ^^^ *p* < 0.001 compared to SQ group; ^###^ *p* < 0.001 compared to DS group).

Sedentary diabetic rats (DS group) had the lowest relaxation along the experiment (*p* < 0.001), compared to the sedentary rats that received quercetin, healthy or with T1DM (S, SQ and DSQ groups), especially at Ach concentrations between 3 × 10^−7^ and 10^−5^ M. Quercetin administration in healthy sedentary rats (SQ group) improved the vessel relaxation only at Ach concentration of 10^−8^ (*p* < 0.01) and of 3 × 10^−5^ M (*p* < 0.001), compared to the healthy rats that did not receive treatment (S group) (Figure 5).

**Figure 6:**
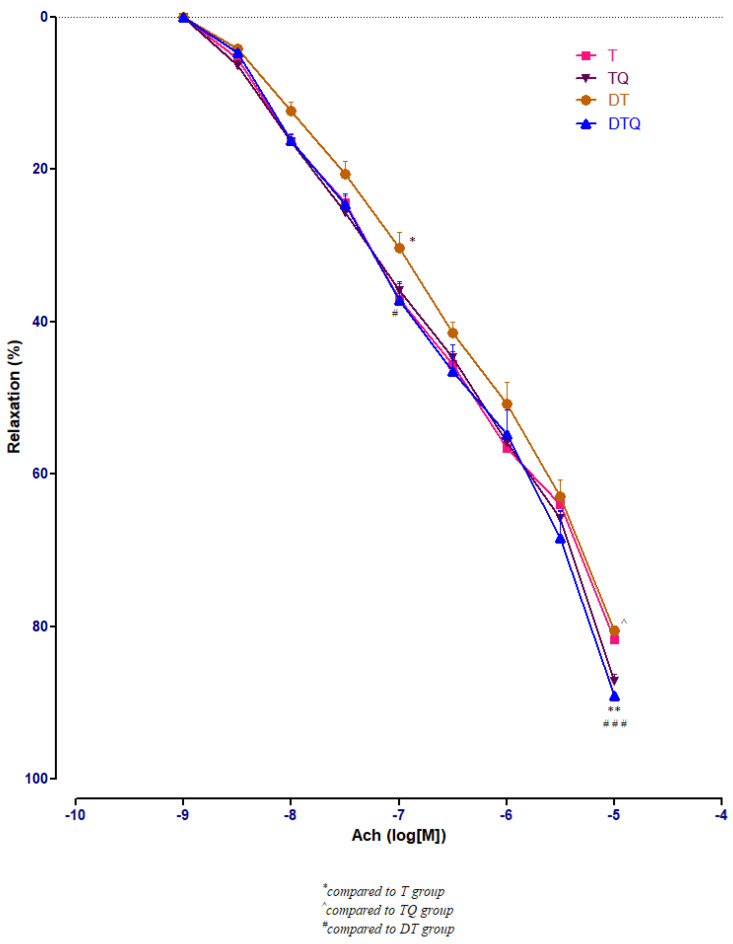
The aorta rings relaxation responses to cumulative concentrations of acetylcholine (Ach) (10^−9^–10^−5^ M) in: trained, untreated animals (T); trained animals, treated with quercetin (TQ); diabetic, trained, untreated animals (DT); diabetic, trained animals, treated with quercetin (DTQ). The relaxation responses are expressed as percentages of relaxation from an induced maximal contraction at PE. The values are expressed as mean ± SEM (* *p* < 0.05, ** *p* < 0.01 compared to T group; ^ *p* < 0.05 compared to TQ group; ^#^ *p* < 0.05; ^###^ *p* < 0.001 compared to DT group).

Quercetin administration improved significantly the vessel relaxation in diabetic trained rats (DTQ group) at 10^−7^ M (*p* < 0.05) and at 10^−5^ M (*p* < 0.001) compared to diabetic trained animals (DT group), and at 10^−5^ M (*p* < 0.01) compared to the healthy trained rats (T group). In diabetic rats (DT group), moderate physical training produced similar aorta responses like in healthy animals (T group) with only one significant modification (*p* < 0.05) recorded at 10^−7^ M Ach concentration (Figure 6). 

**Figure 7:**
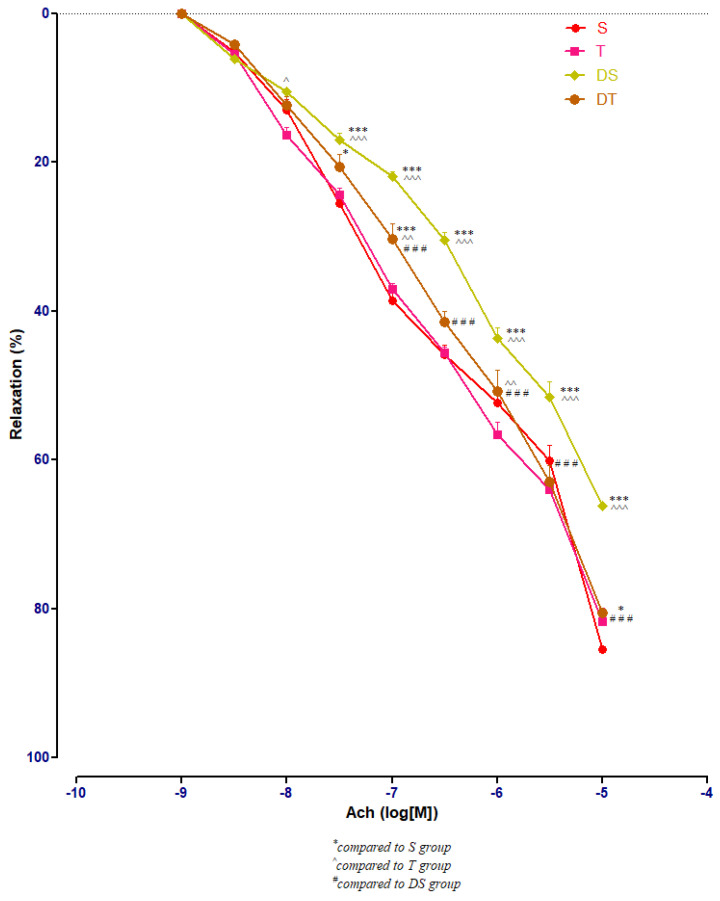
The aorta rings relaxation responses to cumulative concentrations of acetylcholine (Ach) (10^−9^–10^−5^ M) in: sedentary, untreated animals (S); trained, untreated animals (T); diabetic, sedentary, untreated animals (DS); diabetic, trained, untreated animals (DT). The relaxation responses are expressed as percentages of relaxation from an induced maximal contraction at PE. The values are expressed as mean ± SEM (* *p* < 0.05, *** *p* < 0.001 compared to S group; ^ *p* < 0.05, ^^ *p* < 0.01, ^^^ *p* < 0.001 compared to T group; ^###^ *p* < 0.001 compared to DS group).

In healthy rats, moderate swimming training did not modify significantly the aorta rings responses to Ach. In diabetic rats, compared to DS group, moderate physical training (DT group) improved significantly (*p* < 0.001) the aorta relaxation at Ach, at concentrations between 10^−7^ and 10^−5^ M (Figure 7). 

### 3.4. Aortic Relaxation Responses to Sodium Nitroprusside (SNP) in Control and Experimental Groups 

**Figure 8:**
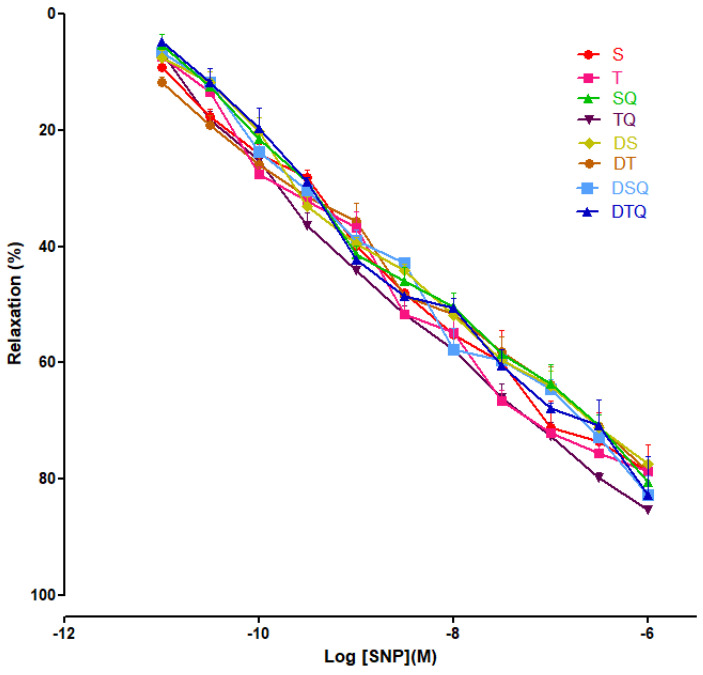
The relaxation responses of the aorta rings, precontracted with phenylephrine (PE), to cumulative concentrations of sodium nitroprusside (SNP 10^−11^ to 10^−6^ M) in: sedentary, untreated animals (S); trained, untreated animals (T); sedentary animals, treated with quercetin (SQ); trained animals, treated with quercetin (TQ); diabetic, sedentary, untreated animals (DS); diabetic, trained, untreated animals (DT); diabetic, sedentary animals, treated with quercetin (DSQ) and in diabetic, trained animals, treated with quercetin (DTQ). The relaxation responses are expressed as percentages of relaxation from an induced maximal contraction at PE. The values are expressed as mean ± SEM.

Moderate swimming training and quercetin effects were evaluated on endothelial-independent relaxation responses to sodium nitroprusside (SNP) (10^−11^ to 10^−6^ M), and not significant modifications were recorded among the investigated groups of rats (Figure 8).

## 4. Discussion

Streptozotocin (STZ) intraperitoneal administration induces type 1 diabetes mellitus (T1DM), a metabolic disease characterised by significant increases of blood glucose levels associated with altered endothelial-dependent relaxation [31,32]. 

The present study showed that, in streptozotocin-induced diabetes mellitus rats, 5 weeks of moderate swimming training associated with quercetin administration significantly reduced the blood glucose levels and restored the endothelial function.

The patients with diabetes mellitus (DM) have a high risk of death because of cardiovascular diseases. The main risk factor involved in cardiovascular diseases development is represented by the endothelial dysfunction [1,7,33]. In T1DM, endothelial dysfunction may be produced by numerous factors including hyperglycaemia, dyslipidemia, decreases of NO bioavailability, insulin resistance or ROS increased synthesis [2,3,34]. Endothelial dysfunction is characterised by altered endothelial-dependent relaxation with satisfactory endothelial-independent vessel relaxation. 

Hyperglycaemia produces endothelial dysfunction through NF-kB (nuclear factor-kB) activation. Therefore, the iNOS (inducible nitric oxide synthase) expression increases, leading to NO increased synthesis. Nitric oxide reacts with superoxide anion radical producing peroxynitrite, a strong oxidant with numerous noxious effects [7,33,34,35].

Streptozotocin is a natural compound produced by Streptomyces achromogenes that produces specific inflammation of pancreatic β-cells with the result of insulin absolute deficit [31,32]. In our experiment, fasting blood glucose (FBG) levels increased significantly after STZ administration. The results of our study showed that quercetin administration (30 mg/kg body weight/day/5 weeks) may produce significant decreases of glycaemia in T1DM. Recent studies revealed the mechanisms through which quercetin exerts its hypoglycaemic effects: it increases insulin sensitivity, inhibits α-glycosidase activity (in vitro), stimulates the hexokinase activity, increases the GLUT-4 (insulin-dependent glucose transporter) mRNA expression and this transporter translocation to the plasma membrane, and stimulates the glycogen production [21,36]. 

In the present study, the diabetic rats with moderate swimming training for 5 weeks showed significant decreased levels of FBG, in comparison with sedentary diabetic rats. These results are concordant to recent studies that presented the hypoglycaemic effects of moderate physical effort in diabetic rats [3,6,11,20,24,37]. The group of rats with DM and moderate swimming training treated with quercetin (DTQ group) showed a greater improvement of FBG levels, compared to the groups with only one approach: only training, or only quercetin treatment. Our results, in concordance with recent studies, may indicate the preservation of β-cells function and GLUT-4 expression as effects of quercetin and moderate swimming training which increase the glucose transport inside the cells [14,37].

In the present study, the vessel response to PE (α_1_-adrenoreceptor agonist) in diabetic rats was investigated. The thoracic aorta rings were precontracted with PE in cumulative doses (10^−9^ to 10^−6^ M). The results of our study showed a significant increase of contractile response in aortas of sedentary diabetic rats, while the quercetin administration or moderate swimming training associated with quercetin treatment decreased significantly the vessels’ contraction. These results may be the effects of endothelial impairment caused by hyperglycaemia, oxidative stress, NO decreased bioavailability, increased synthesis of superoxide anion and dyslipidemia. In our previous study [4], we observed the increased production of ROS, nitrites, and iNOS in diabetic rats’ aortas. In the present study, quercetin administration decreased significantly the levels of mentioned parameters in aorta of diabetic rats while the association of quercetin treatment with moderate physical training had the best effects. 

Acetylcholine produces the relaxation of the vessel smooth muscle layer through an endothelium-dependent mechanism by increasing the synthesis and release of vasodilator substances from this layer, including the NO and prostacyclin. Several studies showed the overreaction to Ach of the diabetic vessels in rats [33,34,35,36], but in our study the diabetic aortas’ response to this neurotransmitter was decreased. The group of rats with DM and moderate swimming training had an improved vascular function. The sedentary and trained diabetic rats treated with quercetin presented an EDR restoring to acetylcholine. These results may be produced in diabetic rats by hyperglycaemia, reduced insulin level in plasma, and by the decrease of NO bioavailability from endothelial cells while the quercetin administration, with or without moderate physical effort training, blocked the NO oxidative inactivation by the superoxide anion. Our findings suggest that quercetin may have antioxidant properties, acting directly on ROS in aorta wall. Recent studies performed on diabetic thoracic aorta showed that endothelial dysfunction is partially determined by the decreased NO release and bioavailability, and by the altered signalling cascades post-NO release from the endothelial cells [36]. Hyperglycaemia induces vessel lesions through different mechanisms including the increase of ROS synthesis, production of advanced glycation end products (AGEs), activation of polyol pathway, and apoptosis. The high levels of plasma glucose increase significantly the ROS synthesis in endothelial cells but also in smooth muscle cells through glucose auto-oxidation, NO decreased synthesis, NO inactivation in a high rate and the increase of PCK activity [37]. 

The mechanisms through which the moderate physical training re-establish the endothelial function in T1DM are still not completely understood. It is presumed that the beneficial effects of moderate exercise training may be produced by the increase of blood flow that increases the shear stress on the endothelium leading to the increased NOS activity and NO bioavailability [4,38]. Moderate exercise training decreases the oxidative stress and the expression of the proinflammatory molecules [38], both mechanisms being considered trigger factors for the endothelial dysfunction, and it also re-establishes the function of the endothelial progenitor cells, promoting the endothelium repair and angiogenesis [38].

Quercetin, a flavonoid found in vegetables and fruits, has numerous favourable effects on health. In diabetes mellitus type 1, quercetin has hypoglycaemic effects through the increase of glucose intracellular transport and glycogen synthesis, decrease of insulin resistance, activation of enzymes involved in glucose metabolism, inhibition of apoptosis and damages through oxidative stress of the β-pancreatic cells, repair of these insulin secreting cells, etc. [39]. Quercetin has antioxidant effects in DM, scavenges the ROS, and also ensures anti-inflammatory and antiapoptotic protection participating in different protective mechanisms [36,39]. The results of the present study related to the quercetin properties in endothelial function recovery are concordant with recent literature data [4,6,36,39].

In our study, SNP (NO donor) did not modify significantly the aorta rings responses, among the groups. 

## 5. Conclusions

Moderate swimming training associated with quercetin administration had hypoglycaemic effects and recovered the aorta reactivity to vasoconstrictor and vasodilator substances in streptozotocin-induced diabetes mellitus, this combination showing better results than their individual effects. The results of our study present the value of combined moderate physical training with quercetin administration in the management of diabetes mellitus type 1.

## Figures and Tables

**Table 1:** The effects of moderate swimming training in association with quercetin on fasting blood glucose and body weights, in experimental groups.

Parameters	S	T	SQ	TQ	DS	DT	DSQ	DTQ
Initial FBG (mg/dL)	69.9 ± 2.23	68.4 ± 1.84	67.6.0 ± 4.6	67.4 ± 3.37	573.1 ± 45.35 ^aaa^	542.8 ± 62.04 ^aaa^	535.5 ± 35.63 ^aaa^	523.0 ± 38.7 ^aaa^
Final FBG (mg/dL)	72.0 ± 3.2	70.0 ± 2.6	69.8 ± 3.6	70.3 ± 2.75	583.1 ± 31.24 ^aaa^	477.4 ± 44.2 ^b^	380.5 ± 25.2 ^bbb^	293.0 ± 38.0 ^bbb^
Initial body weight (g)	271.5 ± 14.6	270.0 ± 16.9	281.4 ± 13.7	288.9 ± 12.9	273.5 ± 18.9	282.5 ± 18.49	279.8 ± 15.27	280.4 ± 18.3
Final body weight (g)	277.5 ± 13.9	265.5 ± 12.9	286.4 ± 11.8	290.0 ± 11.67	238.5 ± 10.34 ^a^	270.9 ± 12.80	274.2 ± 11.03	276.4 ± 13.9

S = sedentary, untreated animals, T = trained, untreated animals, SQ = sedentary animals, treated with quercetin, TQ = trained animals, treated with quercetin, DS = diabetic, sedentary, untreated animals, DT = diabetic, trained, untreated animals, DSQ = diabetic, sedentary animals, treated with quercetin, DTQ = diabetic, trained animals, treated with quercetin. Results are mean ± SD of 10 rats per each group. Statistically significant differences are indicated by the symbols: ^a^ *p* < 0.05, ^aaa^ *p* < 0.0001 vs. S group; ^b^ *p* < 0.05, ^bbb^ *p* < 0.0001 vs. DS group.

## Data Availability

Data is contained within the article.
